# CAR-T Cell Therapy: A Door Is Open to Find Innumerable Possibilities of Treatments for Cancer Patients

**DOI:** 10.4274/tjh.2018.0196

**Published:** 2018-11-13

**Authors:** Lorena Perez-Amill, Berta Marzal, Alvaro Urbano-Ispizua, Manel Juan, Beatriz Martín-Antonio

**Affiliations:** 1 Institut d’Investigacions Biomèdiques August Pi i Sunyer Hospital, Clinic of Hematology, Barcelona, Spain; 2 Institut d’Investigacions Biomèdiques August Pi i Sunyer Hospital, Clinic of Immunology, Barcelona, Spain; 3 Josep Carreras Leukaemia Research Institute, Barcelona, Spain; + Lorena Perez-Amill and Berta Marzal contributed to this article equally.

**Keywords:** CAR-T cell immunotherapy, CD19, BCMA, GD2, HER2, EGFRvIII

## Abstract

Seven years ago a chronic lymphocytic leukemia patient was for the first time successfully treated with chimeric antigen receptor (CAR)-modified T cells (CAR-T cells) to target CD19 overexpression in tumor cells. This was the beginning of the development of a new type of immunotherapy treatment in cancer patients. Since then, identification of novel antigens expressed in tumor cells and optimization of both CAR constructs and protocols of administration have opened up new avenues for the successful treatment of other hematological malignancies. However, research still continues to avoid some problems such as toxicities associated with the treatment and to find strategies to avoid tumor cell immune evasion mechanisms. On the other hand, for solid tumors, CAR-T therapy results are still in an early phase. In contrast to hematological malignancies, the complex tumor heterogeneity of solid tumors has led to the research of novel and challenging strategies to improve CAR-T cell activity. Here, we will review the main clinical results obtained with CAR-T cells in hematological malignancies, specifically focusing on CAR-T-19 and CAR-T against B-cell maturation antigen (CAR-T-BCMA). Moreover, we will mention the main problems that decrease CAR-T cell activity in solid tumors and the strategies to overcome them. Finally, we will present some of the first clinical results obtained for solid tumors.

## Abstract (tr)

Yedi sene önce kronik lenfositik lösemili bir hasta ilk kez başarılı olarak tümör hücrelerinde aşırı sunulan CD19’u hedefleyen kimerik antijen reseptör (CAR)-ile değiştirilmiş T hücreleri (CAR-T hücreleri) ile tedavi edilmiştir. Bu kanser hastalarında yeni bir tip immünoterapinin gelişiminin başlangıcını oluşturmaktaydı. Bunu takiben, tümör hücrelerinde sunulan yeni antijenlerin tanımlanması ve CAR yapılarını ve uygulama protokolleri diğer hematolojik habis tümörlerin başarılı tedavisi için yeni yollar açmıştır. Ancak, tedavi ile ilişkili toksisite gibi bazı problemlerin önlenmesi ve tümör hücresinin immün kaçış mekanizmalarıyla baş edilmesi ile ilgili çalışmalar halen devam etmektedir. Ayrıca, solid tümörler için, CAR-T tedavi sonuçları halen erken dönemdedir. Hematolojik habis tümörlerin aksine, solid tümörlerin karmaşık tümör heterojenitesi CAR-T hücre aktivitesi arttırmaya yönelik yeni ve zorlayıcı stratejilerinin araştırılmasına yol açmıştır. Burada, CAR-T hücrelerinin hematolojik habis tümörlerdeki, özellikle de CAR-T-19 ve B-hücre matürasyon antijenine karşı CAR-T’nin (CAR-T-BCMA) başlıca klinik sonuçlarını gözden geçireceğiz. Ayrıca, solid tümörlerde CAR-T hücre aktivitesini azaltan problemlerden ve bunların üstesinden gelmeye yarayan stratejilerden bahsedeceğiz. Son olarak, solid tümörlerdeki ilk klinik çalışmaların bazılarını sunacağız.

## Introduction: Chimeric Antigen Receptor-T Cell Therapy

The last decade has witnessed a huge increase in new immunotherapy modalities to treat cancer patients, such as the infusion of chimeric antigen receptor (CAR) modified-T cells (CAR-T cells), which represents the most important advance made to treat hematological malignancies in patients with relapsed/refractory (r/r) disease. CARs are composed of different synthetic domains combined into a single functional receptor that provides antigen-binding to an antigen present on the tumor cell and T-cell activation after antigen recognition [1]. Once a specific CAR has been designed, CAR-T cell therapy consists on the ex vivo modification of autologous T cells from the patient to express this CAR on their membranes. Afterwards, CAR-T cells are expanded in vitro for 8-10 days and reinfused into the patient, where they will recognize and kill the tumor cells.

A CAR is composed of three domains: 1) The extracellular region codes for the single-chain variable fragment (scFv) of an antibody against the antigen present in the tumor cell. In this region, there is a spacer/hinge domain derived from CD8 and from immunoglobulin G (IgG) sequences that profoundly affects CAR function and scFv flexibility [2]. 2) The CAR transmembrane domain, derived from T-cell molecules, such as CD3ζ, CD4, CD8a, or CD28, links the extracellular domain with 3) the intracellular domain, which activates the T cells and is composed of CD3ζ T-cell receptor. This is the structure of the first-generation CAR-T cells, which have the benefit of not requiring antigen processing/presentation by the human leukocyte antigen (HLA), allowing them to bypass HLA-I restriction [3,4].

**Figure 1:**
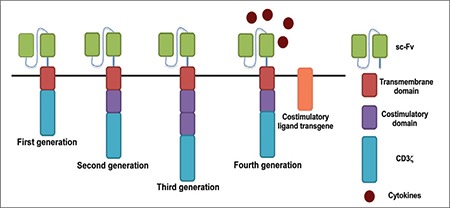
Structure of different chimeric antigen receptor (CAR) generations. First-generation CARs contain the single-chain variable fragment bound to the spacer/hinge domain, a transmembrane domain region with CD8 being the most commonly used, and the T-cell receptor CD3z domain. Second-generation CARs add one costimulatory domain to the construct, and third-generation CARs contain more than one costimulatory domain. Fourth-generation CARs contain an inducible or constitutive domain for another protein such as cytokines or specific ligand receptors. scFv: Single-chain variable fragment.

For the first-generation CAR-T cells, it was observed that even when the CAR-T cell mechanism was active, T cells did not proliferate in vivo, and moreover, a robust cytokine response after recognition of a tumor cell was not observed. This finding led to the addition of costimulatory domains in the CAR construct, giving rise to second- and third-generations CAR-T cells. Initially, CD28 was selected as the costimulatory domain by Savoldo et al. [5], who compared two autologous CAR-T types with the same specificity for CD19, one that encoded CD3ζ and CD28, while the other encoded only CD3ζ. The CAR-T cells containing CD28 showed enhanced expansion and persistence, confirming the requirement of costimulatory domains in the CAR construct. At the same time, Porter et al. [6] observed that the inclusion of 4-1BB as a costimulatory domain increased the antitumor activity and the in vivo persistence of CAR-T cells compared to CAR-T cells with the CD3-ζ domain alone. Therefore, costimulatory domains such as CD28, 4-1BB, and OX40 [7,8,9] were included in second-generation CAR-T cells, providing higher in vivo CAR-T cell proliferation than first-generation CAR-T cells. It was observed that whereas CD28 is better to activate T cells, 4-1BB increases CAR-T cell persistence [10]. Therefore, the majority of recent clinical studies on hematological malignancies are infusing CAR-T cells with 4-1BB. Moreover, third- and fourth-generations of CAR constructs have also been added to the CAR-T arsenal. Third-generation CAR-T cells encode more than one costimulatory domain to enhance T-cell activation and proliferation. Fourth-generation CAR-T cells, also known as TRUCKs or “armored CARs”, incorporate a constitutive or inducible expression domain for a protein that needs to be induced or constitutively secreted. Therefore, these CARs can deliver a product to the targeted tumor tissue (i.e. a cytokine), but they also could incorporate a peptide to recognize and bind to its ligand (i.e. CD40L) in the target cell, and to interact with other immune cells such as dendritic cells (i.e. 4-1BBL) (Figure 1) [11,12].

## Cytokine Release Syndrome Associated with CAR-T Cells

The most common toxicity associated with CAR-T cell therapy is a massive inflammatory response called cytokine release syndrome (CRS), which results from high cytokine levels released after T-cell engagement and proliferation. In most patients, CRS occurs 1-14 days after CAR-T cell infusion. Most patients develop low-grade CRS with fevers and myalgias. However, some patients experience severe CRS with hypotension, pulmonary edema, coagulopathy, vascular leak, and neurotoxicity in some cases, which can result in multiorgan system failure [13]. Interleukin (IL)-6 is a central mediator of CRS and CRS is well managed with tocilizumab, an anti-IL-6 receptor. However, corticosteroids have also been successfully used without compromising CAR-T cell proliferation or efficacy [14,15]. Managing CRS requires performing appropriate grading to define its onset and grading and resolution criteria. Currently, there are three CRS grading scales. The first scale used to define CRS is the one graded by the National Cancer Institute, called the Common Terminology Criteria for Adverse Events; however, this system was not specific for cellular therapeutic approaches. Afterwards, Lee et al. [16] proposed a specific scale for cellular therapeutic modalities, which was slightly modified by the MD Anderson Cancer Center proposing a new grading system [17]. Currently, the most widely used scale is the one proposed by the University of Pennsylvania (UPenn), based on the clinical results of their murine CAR-T-19 (tisagenlecleucel) after treatment of 125 patients with B-cell acute lymphoblastic leukemia (B-ALL) and 42 patients with chronic lymphocytic lymphoma (CLL). This scale is based on easily accessible clinical features and not laboratory values, which makes it possible for it to be applied more widely by many hospitals. It applies to both early-onset and delayed-onset CRS, and it distinguishes between mild, moderate, severe, and life-threatening CRS [18]. This scale was used in two multicenter phase II trials infusing tisagenlecleucel in r/r ALL patients performed in 11 different countries and nine sites in the United States. At all of these centers, using this scale, 81% of the patients experienced some grade of CRS and 45% suffered grade 3 or 4 CRS [18,19]. In addition, this scale has also been adopted for other CAR constructs against B-cell maturation antigen (BCMA) for multiple myeloma (MM) [20] and against mesothelin in epithelial ovarian cancer [21]. Table 1 summarizes the grading and CRS management adopted by our institution.

## From the Initial Stages Infusing CAR-T-19 to a High CAR-T Cell Variety to Treat Different Malignancies

More than 20 years have passed from the first studies with first-generation CAR-T cells [22,23] to the design of second-generation CAR-T cells and finally the first successful clinical study in 2011 to treat a CLL patient with CAR-T-19 cells achieving complete remission (CR) [6]. Since then, an increasing number of clinical studies started to be performed, and today almost 200 clinical trials infusing CAR-T cells are being performed around the world. The greatest results have been obtained with CAR-T-19 in B-cell malignancies. Here, we will review some of the most relevant results obtained with CAR-T-19 and CAR-T-BCMA to treat MM. Moreover, we will mention other CAR constructs employed to treat B-cell malignancies not responding to CAR-T-19.

## CAR-T-19 for the Treatment of B-Cell Malignancies

Three different institutions, the National Cancer Institute (NCI), UPenn, and the Memorial Sloan-Kettering Cancer Center (MSKCC), have been the pioneering centers performing clinical studies infusing second-generation CAR-T-19 cells to treat ALL, CLL, and lymphoma patients. Whereas the NCI and MSKCC have employed CAR-T-19 with CD28 as a costimulatory domain, UPenn selected 4-1BB. Their results have contributed to defining critical parameters including the best costimulatory domain, viral vector, gene transfer method, T-cell stimuli used during T-cell production, conditioning chemotherapy, and T-cell dose [24]. For instance, direct comparison by the MSKCC of CAR-T-19 with and without conditioning chemotherapy showed increased T-cell persistence and improved outcome with conditioning chemotherapy [25]. Regarding T-cell dose, whereas for CAR-T-19 this parameter is not so relevant [24], for other CAR constructs, such as BCMA in MM, a minimal CAR-T cell dose is required to achieve response [26]. In more detail, we will describe some clinical results obtained with CAR-T-19 to treat B-cell malignancies. 

The first treated CLL patient received 1.46x10^5^ CAR-T-19 cells/kg split into three doses. CAR-T cells persisted for 6 months and remission was ongoing for 10 months. Because of this low CAR-T cell dose, CRS was reported 14 days after the first infusion, coinciding with peak levels of CAR-T-19 in peripheral blood (PB) [6]. Afterwards, two pediatric r/r ALL patients were treated with CAR-T-19. The first patient received 1.2x10^7^ CAR-T-19 cells/kg for three consecutive days without lymphodepletion. Patient 2 received 1.4x10^6^ CAR-T-19 cells/kg in a single dose and etoposide-cyclophosphamide was administered the week before. In both patients, expansion of CAR-T-19 was detected, and CR occurred the first month. However, whereas patient 1 had ongoing CR for 11 months, patient 2 had a CD19-negative relapse 2 months after treatment [14]. This was the first study describing CD19-negative relapses, one of the main problems after CAR-T-19 immunotherapy, which occurs in 78% of relapsed patients [27]. This year updated results on 75 children and young adults receiving CAR-T-19 (tisagenlecleucel) to treat ALL have shown overall response (OR) of 81% within 3 months, including 60% CR. CRS occurred in 77% of patients [27]. These results provided the basis for the approval of the first gene therapy product in the United States in 2017, tisagenlecleucel, commercialized by Novartis to treat B-cell precursor ALL patients up to 25 years old [28].

Recently, the MSKCC published results for CAR-T-19 with CD28 in 53 adult r/r ALL patients. At 29 months 83% CR was obtained, while median disease-free survival (DFS) and overall survival (OS) were 6.1 and 12.9 months, respectively. Severe CRS occurred in 26% of patients. Patients with low disease burden showed higher remission rates with 20.1 and 10.6 months of OS and DFS, respectively, and lower CRS than patients with higher disease burden [29].

Whereas results in ALL have been remarkable, in CLL and lymphoma cases the clinical results have been poorer. Comparison of 14 phase I clinical trials between 1991 and 2014 including 119 patients demonstrated that the OR rate was 73%, with 93% of responses in ALL patients, followed by CLL with 62% and lymphoma patients with 36%. Moreover, lymphodepletion, higher CAR-T cell dose, and no interleukin (IL)-2 administration were associated with better responses [30]. Interestingly, a more recent study of 24 CLL patients showed that CAR-T-19 is highly effective in high-risk CLL relapsed patients after ibrutinib treatment, showing OR of 71% and 83% CRS [31].

The CAR-T-19 from UPenn was used in 28 patients with r/r diffuse large B-cell lymphoma (DLBCL) or follicular lymphoma with CAR-T cell doses from 1x10^8^ to 5x10^8^. CRS occurred in 18% of patients while 90% CR was obtained after 1 month. The CR rate at 3 months was 43% and 71% in DLBCL and follicular lymphoma patients, respectively. At 28.6 months, sustained remissions were maintained in 86% of DLBCL and in 89% of follicular lymphoma patients [32]. 

On the other hand, the CAR-T-19 from the NCI with CD28 was employed in a phase I study of 7 patients with r/r DLBCL. Patients received 2x10^6^ CAR-T-19 cells/kg. One patient (14%) experienced grade 4 CRS. Grade ≥3 CRS and neurotoxicity were observed in 14% and 57% of patients, respectively. OR and CR were 71% and 57%, respectively. At 12 months, 43% of patients remained in CR [33]. Based on these results, a multicenter phase 2 study was performed to treat 101 r/r patients with DLBCL, primary mediastinal B-cell lymphoma, or transformed follicular lymphoma. Patients received 2x10^6^ CAR-T-19 cells/kg. Grade 3 or higher CRS and neurologic events occurred in 13% and 28% of the patients, respectively. OR was 82% and CR was 54%. At 15.4 months, 42% of the patients continued having a response, with a 40% rate of CR. OS at 18 months was 52%. Of the patients who showed disease progression, 27% of them showed CD19-negative disease [34]. Based on these results, the Food and Drug Administration approved the first CAR-T-19 cell product, called axicabtagene ciloleucel (Yescarta, Kite Pharma), to treat DLBCL, primary mediastinal large B-cell lymphoma, and high-grade B-cell lymphoma [35].

## Other CAR Constructs Employed to Treat B-Cell Malignancies Not Responding to CAR-T-19

Other CAR constructs, such as CAR-T cells against CD30 (CAR-T-30), have been used to treat to treat Hodgkin lymphoma (HL) and anaplastic large cell lymphoma (ALCL), which do not express CD19. Recently, the induction of CR in 9 r/r patients with HL (7 patients) and ALCL (2 patients) even in the absence of a conditioning regimen was reported without CAR-related toxicities. Patients received from 0.2x10^8^ to 2x10^8^ of CAR-T-30 cells/m^2^. Seven of 9 patients received two or more infusions of CAR-T-30. Fourteen percent of HL patients entered CR lasting more than 2.5 years after the second infusion, 14% remained in CR for almost 2 years, and 43% had transient stable disease. For ALCL, one patient had CR for 9 months after the fourth infusion of CAR-T cells. Interestingly, although CD30 may be expressed by normal activated T cells, no patients developed impaired virus-specific immunity [36]. Tables 2 and 3 summarize additional studies of other targets.

## CAR-T-BCMA for MM and Other B-Cell Malignancies

BCMA has appeared as a promising target to treat MM patients due to specific BCMA expression in plasma cells and its absence in most tissues [37]. Currently, more than 20 clinical trials are infusing CAR-T-BCMA for MM treatment. Due to the restricted BCMA expression pattern, BCMA was defined as the most suitable antigen to treat MM, and the design of novel and effective CAR-T-BCMA with CD28 [38] opened the path for a clinical trial in MM patients in 2016. This study infused CAR-T-BCMA in 12 r/r MM patients. Patients received different CAR-T-BCMA cell doses (0.33x10^6^, 1x10^6^, 3x10^6^, and 9x10^6^ CAR-T-BCMA cells/kg). The 2 lowest doses achieved limited responses. At the third dose, a partial loss of BCMA expression in MM cells was detected in one patient, and one patient (25%) obtained very good partial response (VGPR). At the highest dose, one patient (50%) achieved CR for 17 weeks before relapse, and the other patient showed VGPR for 28 weeks. Both patients developed CRS [26]. These results were extended to perform a multicenter study to treat 21 patients in a dose-escalation study. CD28 was changed by 4-1BB and the CAR-T-BCMA was now called bb2121. It was found that 71% of patients developed CRS. The lowest dose (50x10^6 ^cells) infused in three patients was not active. The other 18 patients receiving 150x10^6^ (6 patients), 450x10^6^ (9 patients), and 800x10^6^ cells (3 patients) showed 94% OR, 89% VGPR, and 56% CR. Durable responses were ongoing over 1 year, and more importantly, responses continued to improve over time from VGPR to CR [39].

Additional studies with CAR-T-BCMA have been also successful. Cohen et al. [40] treated 21 r/r MM patients with CAR-T-BCMA in split-doses (10% on day 0, 30% on day 1, and 60% on day 2). Patients were assigned to three cohorts: 1-5x10^8^ CAR-T cells (cohort 1: 9 patients), cyclophosphamide (CXT) 1.5 g/m^2^ + 1-5x10^7^ CAR-T cells (cohort 2: 5 patients), and CTX 1.5 g/m^2^ + 1-5x10^8^ CAR-T cells (cohort 3: 7 patients). Cohort 1 showed the highest CRS at 89% and 1 patient had ongoing CR at 21 months. Cohorts 2 and 3 showed 75% CRS. Cohort 2, with the lowest CAR-T dose, showed the lowest response (40%), which progressed at 4 and 2 months. Cohort 3, with a high CAR-T dose, at 1 month of follow-up showed 83% of any type of response. Interestingly, in 83% of the patients with ≥PR, MM cells showed decreased BCMA intensity [40]. More recently, a human CAR-T-BCMA was developed at the MSKCC, which hopefully will avoid early disappearance of CAR-T cells. Clinical results with this human CAR construct were recently published [41]. At our institution (Hospital Clinic), we have designed a highly effective CAR-T-BCMA. Moreover, we have also humanized the scFv, confirming its high efficacy, and in the next few months it will be used in a multicenter phase I study to treat r/r MM patients. 

Moreover, Friedman et al. [42], who designed the CAR-T-BCMA (bb2121) [26], identified BCMA expression in primary lymphoma and CLL cells and confirmed the high efficacy of CAR-T-BCMA against models of MM, Burkitt lymphoma, and mantle cell lymphoma, suggesting that this CAR construct could be also efficient for these malignancies.

One of the problems observed in CAR-T immunotherapy for MM is the proportion of relapsed patients no longer having BCMA expression. Different options to avoid this, such as the use of dual CAR constructs targeting two different antigens, are being tested. Lee et al. [16] confirmed that 100% of primary MM cells expressed BCMA, and 78% of them also expressed TACI. Therefore, they successfully tested a third-generation dual CAR-T-APRIL (a ligand for BCMA and TACI), which eliminated MM cells expressing either BCMA or TACI and demonstrated tumor control in the absence of BCMA [43].

The impressive results in r/r MM patients targeting BCMA [39] suggest that after CAR-T-19, BCMA will be the next area where CAR-T therapy will have a high clinical impact. However, some problems still need to be addressed, such as the high CAR-T cell dose required to achieve responses, which could cause high CRS rates. New clinical protocols will aim to ameliorate severe CRS. Interestingly, for other CAR constructs such as CAR-T-20, Watanabe et al. [44] observed that the threshold of antigen density in the tumor required to induce CAR-T cell lytic activity was around 200 molecules per target cell, and for cytokine production it was 10-fold higher, suggesting a range for antigen density in the tumor cell where cytotoxicity can be performed without development of CRS. Second, the loss of BCMA expression in MM cells after CAR-T-BCMA treatment is a problem. Different strategies, such as dual CAR constructs, are being tested. Third, early disappearance of CAR-T cells in patients may be solved with the use of human and humanized CARs.

## Homemade CARs: A Reality?

As previously mentioned, CAR-T-19 cell products have been commercialized by pharmaceutical companies, with prices of $475,000 for tisagenlecleucel and $373,000 for Yescarta. If the positive results obtained continue this trend, hopefully CAR-T-BCMA will also be approved for use in MM patients. Unfortunately, these prices are not affordable for many public national health systems. In this sense, at our institution, we have manufactured our CAR-T-19 cell product. This process requires having a good manufacturing practice facility to perform the viral production. Afterwards, the T-cell transfection is performed in the Prodigy device (Miltenyi, Biotec), a sterile isolated system, which performs all the steps required, starting from the apheresis product to the final product of CAR-T cells. This option provides much more affordable prices that can be assumed by a public national health system. With these CAR-T-19 cells (called ARI-0001), 18 patients with r/r B-cell malignancies have already been treated and a phase II clinical trial is about to start.

## CAR-T Cells for the Treatment of Solid Tumors

Contrary to hematological malignancies, severe side effects, lack of persistence and effectiveness of CAR-T cells, immunosuppression in the tumor microenvironment, lack of homing, and tumor-off/target-on effects occurring in solid tumors decrease the success of CAR-T therapy for these malignancies [45]. Some strategies employed to improve these problems include the following: 1) Fourth-generation CAR-T cells, by incorporating additional features, such as costimulatory ligands next to the CAR receptor and more than one costimulatory domain, improve the lack of persistence and efficacy of CAR-T cells. In this sense, combining CD28 with OX40 blocks IL-10 production, increasing persistence and conferring higher efficacy to the CAR-T cells [45,46,47]. Combination of CAR-T cells with oncolytic viruses has also been suggested to improve CAR-T efficacy [48]. 2) To overcome the immunosuppressive microenvironment, the preselection of virus-specific CTLs before CAR-T cell transduction achieves a double CAR-T stimulation, either by the TCR or by the CAR, appearing as an option to avoid loss of expression of the tumor antigen [49]. Another option being tested is the combination of CAR-T cells with immunocheckpoint inhibitors, which seems to improve the potency of CAR-T cells [47]. In addition, fourth-generation CAR-T cells can modulate the tumor environment through the secretion of IL-12 and can also increase tumor cell-CAR-T cell contact by the release of adhesion molecules or enzymes that degrade the extracellular matrix [50,51]. CRISPR/CAS9 technology appears as a further option to generate CAR-T cells resistant to exhaustion and inhibition [52]. 3) Moreover, the high tumor-off/target-on effect occurring in solid tumors can be ameliorated by variations in the administration route for CAR-T cells, cell dose, reduction of scFv affinity, use of “switchable CARs”, and the discovery of specific tumor-associated antigens [45,53]. Additional proposals for CAR construct design include the insertion of caspase 9 into the CAR construct, which after administration of a small molecule (AP1903) to the patient will induce apoptosis of 99% of CAR-T cells [54]. Inducible caspase 9 is already being used in clinics, demonstrated to be safe (Table 4). Moreover, the design of transient CAR-T cells by introducing CAR-T mRNA by electroporation has shown antitumor activity in CAR-T-19 for CLL patients [55] and CAR-T-mesothelin for solid tumors [56], and it is being employed in clinical trials (Table 4). Due to all these limitations, positive clinical results with CAR-T cells in solid tumors are scarce, most of them in phase I trials. We will now mention some of the most interesting results obtained with CAR-T cells in solid tumors.

Specific disialoganglioside 2 (GD2) expression in tumor cells and slight expression in normal cells [57] makes GD2 a good candidate for CAR-T therapy, specifically for neuroblastoma. Eight neuroblastoma patients receiving Epstein-Barr virus (EBV)-virus-specific CTLs with CAR-T-GD2 showed evidence of tumor necrosis and one patient remained in CR, suggesting that virus-specific CTLs expressing CAR-T-GD2 show higher persistence in contrast to virus-nonspecific CAR-T cells [49].

Human epidermal growth factor receptor 2 (HER2) is not detected in normal brain tissues, being overexpressed in 25%-30% of breast and ovarian cancers, 60% of osteosarcomas, 80% of glioblastoma multiforme (GBM) cases, and 40% of medulloblastomas [51]. Although HER2 has been successfully targeted with anti-HER2-antibodies (trastuzumab and pertuzumab) in HER2/neu2+ breast cancer, the first breast cancer patient treated with CAR-T-HER2 died because of severe toxicity related to tumor-off/target-on effect [58]. In contrast, gliomas, glioblastomas, GBM, and medulloblastomas showing lower levels of HER2 than breast cancer are not efficiently treated with trastuzumab. Therefore, 17 GBM patients received from 10^6^ to 10^8^ cells/m^2^ of intravenous polyclonal EBV-cytomegalovirus and adenovirus-specific T cells transduced with CAR-T-HER2 (CAR-T-FRP5). Median OS was 11 months, no serious side effects were reported, and CAR-T cells were detected in PB 12 months later [59]. 

IL-13 receptor alpha-2 (IL-13Ra2) is overexpressed in 75% of glioblastoma patients [60,61,62]. The first study in 3 glioblastoma patients receiving up to 12 local intracranial infusions of virus-specific CTL clones transduced with CAR-IL-13Ra2 (E13Y-zetakine CAR) showed minimal side effects and transient responses in 2 patients [63]. Afterwards, the CAR construct was modified to incorporate 4-1BB and a mutated IgG4-Fc linker to reduce tumor-off/target-on effect. At a dose of 2x10^6^ these CAR-T cells were administrated by intracranial infusion to one glioblastoma patient, followed by five additional infusions of 10x10^6^ CAR-T cells. Severe toxicities did not develop and regression of intracranial and spinal tumors during 7.5 months was observed [64].

Most GBM patients overexpress the mutated epidermal growth factor receptor (EGFR) variant III (EGFRvIII), which is associated with tumor progression and poor prognosis [65]. Comparison of humanized second- and third-generation CAR-T cells with 4-1BB and/or CD28/4-1BB against EGFRvIII in vitro and in vivo demonstrated higher efficacy for the third-generation CAR-T cells. Moreover, a lower-affinity scFv was designed to minimize the tumor-off/target-on effects, and finally this CAR-T cell combined with temozolomide was the optimal strategy in a xenograft glioblastoma model [66]. Based on these results, UPenn conducted the first study with 10 newly diagnosed patients with recurrent GBM with residual disease infusing intravenous CAR-T-EGFRvIII cells. No evidence of off-tumor toxicity or CRS was observed. One patient had residual stable disease for over 18 months. All patients demonstrated transient expansion and trafficking of CAR-T cells to regions of active GBM. However, expression of inhibitory molecules and regulatory T-cell infiltration after CAR-T-EGFRvIII infusion was detected in the tumor environment [67]. Many other ongoing clinical studies targeting EGFRvIII, GD2, and HER2 are summarized in Table 5.

## Conclusion

In summary, CAR-T immunotherapy has achieved remarkable results in the treatment of hematological malignancies, leading to the commercialization of CAR-T cells as pharmaceutical products. Despite positive results, problems such as loss of expression of the target antigen and CRS could be improved. In solid tumors, additional complications due to intratumoral cell heterogeneity cause low responses and high toxicities. Novel CAR designs, modification of clinical protocols, discovery of novel tumor-specific antigens, and novel molecular strategies will improve clinical results for both hematological and solid tumors.

## Figures and Tables

**Table 1:**
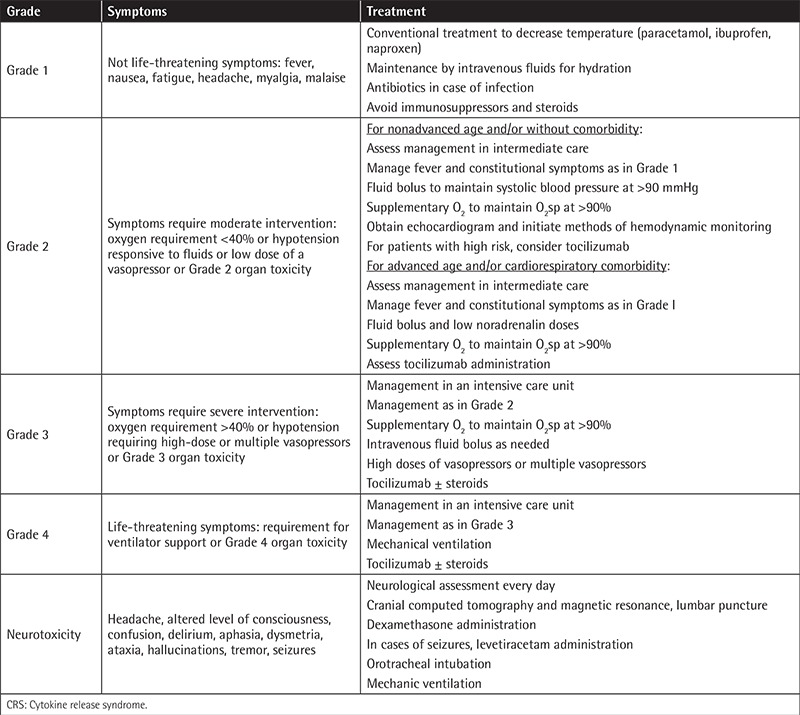
Grading of cytokine release syndrome and management of complications performed at our institution (Hospital Clinic of Barcelona) based on the grading scales of Lee et al. [16] and UPenn Porter et al. [18] and management recommendations.

**Table 2:**
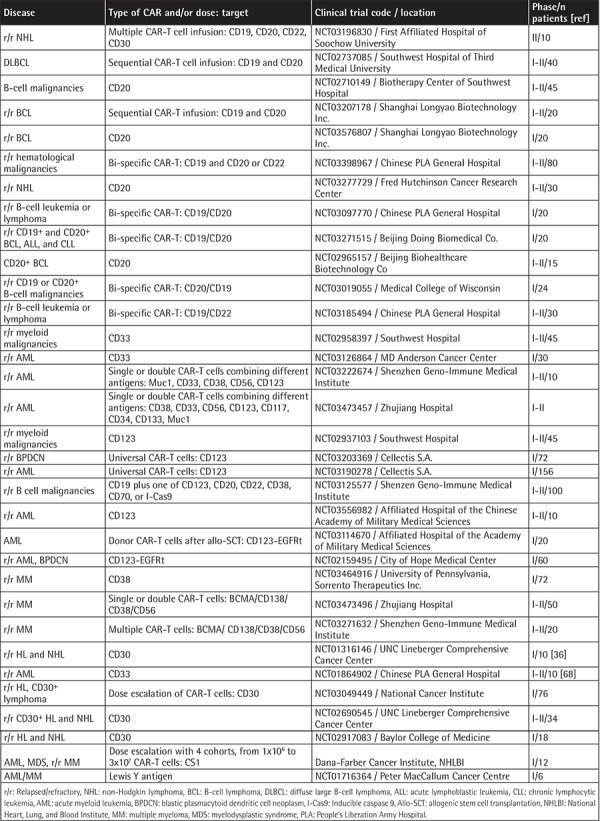
Clinical trials ongoing at other institutions other than the National Cancer Institute, University of Pennsylvania, and Memorial Sloan Kettering Cancer Center targeting CD19, CD20, and CD22 for B-cell malignancies, and other targets in other hematological malignancies.

**Table 3:**
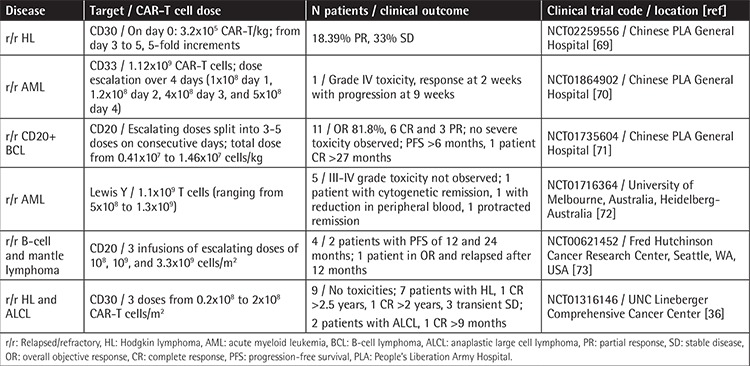
Clinical studies infusing CAR-T cells published by other institutions than the National Cancer Institute, University of Pennsylvania, and Memorial Sloan Kettering Cancer Center.

**Table 4:**
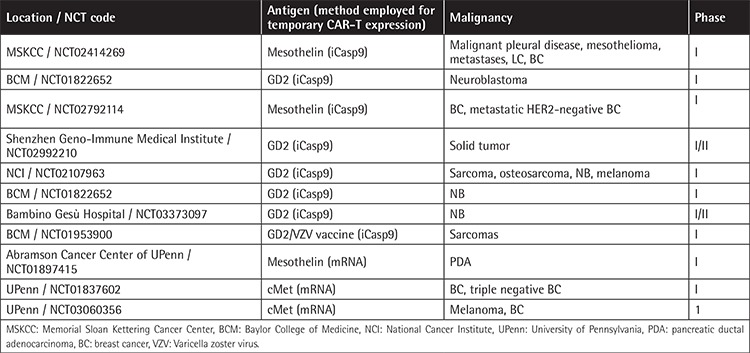
Clinical trials incorporating inducible caspase 9 in CAR-T cells or performing mRNA electroporation to induce the CAR.

**Table 5:**
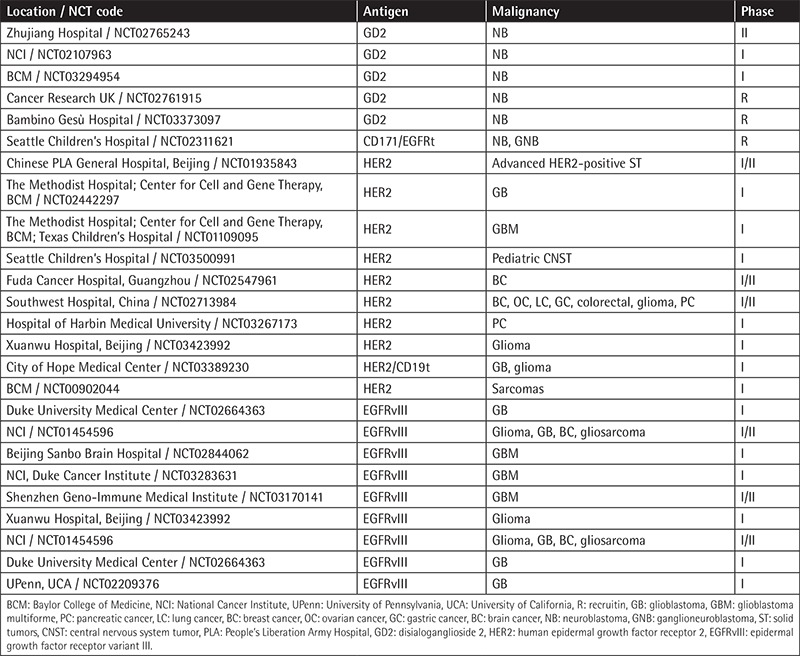
Clinical trials targeting disialoganglioside 2, human epidermal growth factor receptor 2, and epidermal growth factor receptor variant III.
